# Wealth and sexual behaviour among men in Cameroon

**DOI:** 10.1186/1472-698X-6-11

**Published:** 2006-09-11

**Authors:** Eugene J Kongnyuy, Charles S Wiysonge, Robinson E Mbu, Philip Nana, Luc Kouam

**Affiliations:** 1 Department of Obstetrics and Gynaecology, Faculty of Medicine and Biomedical Sciences, University of Yaoundé I, Yaoundé, Cameroon; 2 Liverpool School of Tropical Medicine, Liverpool, UK; 3 Department of Medicine, Faculty of Health Sciences, University of Cape Town, Cape Town, South Africa

## Abstract

**Background:**

The 2004 Demographic and Health Survey (DHS) in Cameroon revealed a higher prevalence of HIV in richest and most educated people than their poorest and least educated compatriots. It is not certain whether the higher prevalence results partly or wholly from wealthier people adopting more unsafe sexual behaviours, surviving longer due to greater access to treatment and care, or being exposed to unsafe injections or other HIV risk factors. As unsafe sex is currently believed to be the main driver of the HIV epidemic in sub-Saharan Africa, we designed this study to examine the association between wealth and sexual behaviour in Cameroon.

**Methods:**

We analysed data from 4409 sexually active men aged 15–59 years who participated in the Cameroon DHS using logistic regression models, and have reported odds ratios (OR) with confidence intervals (CI).

**Results:**

When we controlled for the potential confounding effects of marital status, place of residence, religion and age, men in the richest third of the population were less likely to have used a condom in the last sex with a non-spousal non-cohabiting partner (OR 0.43, 95% CI 0.32–0.56) and more likely to have had at least two concurrent sex partners in the last 12 months (OR 1.38, 95% CI 1.12–1.19) and more than five lifetime sex partners (OR 1.97, 95% CI 1.60–2.43). However, there was no difference between the richest and poorest men in the purchase of sexual services. Regarding education, men with secondary or higher education were less likely to have used a condom in the last sex with a non-spousal non-cohabiting partner (OR 0.24, 95% CI 0.16–0.38) and more likely to have started sexual activity at age 17 years or less (OR 2.73, 95% CI 2.10–3.56) and had more than five lifetime sexual partners (OR 2.59, 95% CI 2.02–3.31). There was no significant association between education and multiple concurrent sexual partnerships in the last 12 months or purchase of sexual services.

**Conclusion:**

Wealthy men in Cameroon are more likely to start sexual activity early and have both multiple concurrent and lifetime sex partners, and are less likely to (consistently) use a condom in sex with a non-spousal non-cohabiting partner. These unsafe sexual behaviours may explain the higher HIV prevalence among wealthier men in the country. While these findings do not suggest a redirection of HIV prevention efforts from the poor to the wealthy, they do call for efforts to ensure that HIV prevention messages get across all strata of society.

## Background

The toll of the acquired immunodeficiency syndrome (AIDS) pandemic has outstripped the worst predictions, especially in sub-Saharan Africa which is home to two-thirds of all people living with the human immunodeficiency virus (HIV) worldwide [1]. There has been an intense debate in the last four years on the relative roles of unsafe sex and unsafe health care on HIV spread in sub-Saharan Africa [2-7], but most public health experts believe that sexual transmission is the dominant mode of HIV spread in the region [1,8]. Risk factors for sexual transmission of HIV include multiple concurrent sexual partnerships, commercial sex, and inconsistent or non-use of condoms. These and other practices are influenced by many factors including lack of accurate information on the modes of HIV transmission, ignorance of own or sex partner's HIV status, culture, economic conditions, mobility, and gender inequalities [9-12].

Economic and social conditions are significantly associated with HIV infection [13-15]. Kalichman and colleagues found that in South Africa, poor education, unemployment, discrimination, violence, and crime were significantly associated with HIV infection [16]. Poverty-related stressors have also been reported as strong predictors of a history of injecting drug use and deviant sexual behaviour, which are all significant risk factors for HIV infection [17]. However, findings have not been consistent throughout the literature. Studies in Kenya and Tanzania have shown HIV infection to be more prevalent among the rich than the poor people [18], and Fenton has questioned whether reducing poverty could lead to a sustainable solution to the HIV pandemic [19]. In addition, evidence from other investigations suggest that the rich may have a high risk of HIV infection because of exposure to unsafe injections given their (potential) greater access to health services [2-4,20-22]. While wealthy men may have a high risk of HIV infection due to disposable income, mobility, etc, poor women are more vulnerable to HIV infection due to lack of prevention choices and sale of sexual services within relationships for survival or advancement in harsh conditions [23,24]. Thus, the exact relationship between wealth and HIV infection remains blurred after more than two decades of research.

The 2004 Cameroon Demographic and Health Survey (DHS) showed the prevalence of HIV in the adult population in the country to be about 5.5%, significantly higher in wealthy people than their poorer counterparts [25]. HIV prevalence was 6.6% among people in the richest quintile of the population compared to 2.4% in the poorest quintile. In addition, it was 6.0% in people with secondary or higher education compared to 3.2% in those who have never been to school. It is not certain whether the higher prevalence results from wealthier people adopting more unsafe sexual behaviours, surviving longer due to greater access to treatment and care, or being exposed to unsafe health care and other HIV risk factors. Since the Joint United Nations Programme on HIV/AIDS and the World Health Organization state that unsafe sex is the main driver of the HIV epidemic in sub-Saharan Africa [1], the aim of this study is to examine the association between wealth and sexual behaviour among men in Cameroon.

## Methods

### Study design

This cross-sectional study is based on data from the 2004 DHS. The survey was approved by the Ethics Committee of the ORC Macro at Calverton in the USA and by the National Ethics Committee in the Ministry of Health in Cameroon. All study participants gave informed consent before participation and all information was collected confidentially.

### Sampling technique

Methods used in the Cameroon DHS have been published elsewhere [25]. Briefly, the survey used a two-stage cluster sampling technique. The country was stratified into 12 domains (10 provinces and 2 major cities). Each domain is made up of enumeration areas (EAs) established by a general population and housing census in 2003. The sampling frame was a list of all EAs (clusters). Within each domain, a two-stage sample was selected. The first stage involved selecting 466 clusters (primary sampling units) with a probability proportional to the size, the size being the number of households in the cluster. The second stage involved the systematic sampling of households from the selected clusters. All men aged 15 to 59 years in the selected households were interviewed.

### Data collection

Data were collected by visiting households and conducting face-to-face interviews to obtain information on demographic characteristics, wealth, and sexual behaviour; among other data. For the current study we extracted information on the 4409 sexually active men who participated in the survey.

### Variables

We extracted data on sexual behaviour, wealth, age, place of residence, marital status, and religion.

We used five characteristics to define sexual behaviour: (a) age at first sexual activity (17 years or less versus more than 17 years), (b) condom use during the last sex with a non-spousal non-cohabiting partner (no versus yes), (c) number of sex partners in the last 12 months (2 or more versus 1 or none), (d) number of lifetime sex partners (more than 5 versus 5 or less), (e) ever paid for sex (yes or no). The cut-off points for age at coital debut and number of lifetime sex partners are based on the median values of Cameroonian men. In this study, unsafe sexual behaviour refers to behaviours that put people at risk of sexual transmission of HIV such as early onset of sexual activity, unprotected sex, multiple concurrent sex partners, multiple lifetime sex partners, and commercial sex.

Two variables were measured as proxy measures of wealth, that is, the wealth index and level of education attained. A score was attributed to each household amenity and the total score constituted the wealth index score [26]. We divided this score into three equal classes of wealth based percentiles, that is, less than 33.33th percentile (low, i.e. poorest), 33.33 to 66.66th percentile (medium, i.e. moderately rich), and more than 66.66th percentile (high, i.e. richest). The level of education attained was defined as never been to school, primary, and secondary or higher education.

Other variables were defined as follows: age was stratified into three 15 year age bands (15–29 years, 30–44 years, and 45–59 years), place of residence was defined as rural or urban, religion (was stratified into Christians, Muslims, and others), and marital status (was defined as never married, currently married, and divorced or widowed).

### Statistical analyses

All cases in the DHS data are given weights to adjust for differences in probability of selection of subjects and to adjust for the non-response in order to produce the proper representation of the whole country [25]. The weight is determined such that it is inversely proportional to the response rate as well as the probability of selection. Therefore, the use of weights corrects for the differential response rates and the unequal probability used to select subjects in the sample. Data were collected and analysed using SPSS version 13.0 for Windows. We used individual weights data analysis in this study. Values for categories of the socio-demographic variables are expressed as absolute numbers (proportions). Unadjusted logistic regression analyses were carried out to investigate the bivariate relationship between each socio-demographic variable and sexual behaviour. Multiple logistic regression analyses were then carried out to find out which of the characteristics were independently associated with sexual behaviour. In the logistic regression models, the dependent variables were sexual behaviours (age at sexual debut, condom use in last casual sex, purchase of sexual services, number of sex partners in the previous 12 months, and number of lifetime sex partners) and the independent variables were wealth index, educational level, age, marital status, religion, and place of residence. The significance tests were two-tailed and statistical significance was defined at the alpha level of 0.05.

## Results

### Socio-demographic characteristics of study participants

A total of 4409 sexually active men participated in the study. Their mean age was 32.4 (standard deviation [SD] 11.4) years and the mean age at first sex was 18.0 (SD 4.1) years. The socio-demographic characteristics of the study population are shown in Table 1. Most of the study population was quite young, with the age group 15–29 years making up 48.3%. A majority of the men were married (60.5%) and the rest had never married (28.7%) or were divorced or widowed (10.9%). More than half (52.1%) had at least secondary education and 56% have had more than five lifetime sex partners.

**Table 1:** Socio-demographic characteristics of 4409 Cameroonian men who participated in the study, 2004

**Characteristic**	**Number (percentage)**^a^
***Age group (in years)***	
15 – 29	2137 (48.4)
30 – 44	1475 (33.4)
45 – 59	808 (18.3)
***Marital status***	
Never married	1267 (28.7)
Currently married	2671 (60.5)
Widow or divorcee	482 (10.9)
***Place of residence***	
Rural	1864 (42.2)
Urban	2556 (57.8)
***Religion***	
Christians	3096 (70.0)
Muslims	755 (17.1)
Others	569 (12.9)
***Level of education***	
No school	540 (12.2)
Primary	1573 (35.6)
Secondary/higher	2306 (52.2)
***Wealth index***	
Low	1456 (32.9)
Middle	1463 (33.1)
High	1501 (34.0)
***Number of lifetime sex partners***	
0 – 5	1937 (43.8)
6 – 95	2473 (56.0)
***Age at first sexual intercourse***	
≤ 17 years	2192 (49.6)
> 17 years	2215 (50.1)

^a^Percentages may not add up to 100% because of missing values.

### Univariate analyses

Bivariate associations between socio-demographic characteristics of the study population, including wealth, and sexual behaviour are shown in Table 2. Compared to poor men, the wealthiest men were more likely to start sexual activity at 17 years or less (OR 1.84, 95% CI 1.59 to 2.13) and less likely to have used a condom in the last sex with a non-spousal non-cohabiting partner (OR 0.16, 95% CI 0.13 to 0.19). In addition, the richest third of the study population were more likely to have had at least two concurrent sex partners in the last 12 months (OR 1.40, 95% CI 1.22 to 1.62) and more than five lifetime partners (OR 1.66, 95% CI 1.45 to 1.90). Wealth was not significantly associated with transactional sex. Compared with those who had never been to school, men who had secondary or higher education were more likely to have started sex at 17 years or less (OR 4.88, 95% CI 3.91 to 6.06) and less likely to have used a condom in the last sex with a non-spousal non-cohabiting partner (OR 0.8, 95% CI 0.06 to 0.12). In addition, the more educated men were more likely to have had at least two concurrent sex partners in the last 12 months (OR 1.32, 95% CI 1.08 to 1.60) and more than five lifetime sex partners (OR 2.02, 95% CI 1.68 to 2.43). The association between educational level attained and purchase of sexual services was not consistent.

**Table 2:** Unadjusted odds ratios of the associations between selected characteristics and sexual behaviours

**Characteristic**	**Had at least two sex partners in the last 12 months (Yes/No)**	**Had more than five lifetime sex partners (Yes/No)**	**Used condom in last sex with non-spousal non-cohabiting partner (Yes/No)**	**Has ever paid for sex (Yes/No)**	**Coital debut at 17 years or younger (Yes/No)**
*Wealth index*					
Low	1	1	1	1	1
Middle	1.09 (0.94–1.26)	1.39 (1.22–1.59)	0.30 (0.24–0.36)	1.26 (0.78–2.04)	1.52 (1.31–1.75)
High	1.40 (1.22–1.62)	1.66 (1.45–1.90)	0.16 (0.13–0.19)	0.88 (0.54–1.41)	1.84 (1.59–2.13)
p-value	0.009	<0.001	<0.001	0.057	<0.001
*Education*					
No school	1	1	1	1	1
Primary	0.94 (0.77–1.15)	1.65 (1.36–2.00)	0.24 (0.16–0.37)	2.25 (1.04–4.88)	3.10 (2.47–3.88)
Secondary/higher	1.32 (1.08–1.60)	2.02 (1.68–2.43)	0.08 (0.06–0.12)	1.47 (0.70–3.09)	4.88 (3.91–6.06)
p-value	0.010	<0.001	<0.001	0.029	<0.001
*Age (years)*					
15–29	1	1	1	1	1
30–44	1.71 (1.50–1.96)	5.58 (4.70–6.62)	3.56 (3.03–4.18)	0.89 (0.59–1.34)	0.39 (0.34–0.45)
45–59	1.32 (1.11–1.56)	4.43 (3.88–5.06)	9.28 (7.04–12.24)	0.17 (0.06–0.47)	0.20 (0.17–0.25)
p-value	<0.001	<0.001	<0.001	0.154	<0.001
*Residence*					
Rural	1	1	1	1	1
Urban	1.18 (1.05–1.33)	1.27 (1.14–1.42)	0.31 (0.26–0.36)	1.45 (0.97–2.18)	1.37 (1.22–1.54)
p-value	0.019	0.001	<0.001	0.966	<0.001
*Religion*					
Christians	1	1	1	1	1
Muslims	0.69 (0.59–0.82)	0.47 (0.40–0.54)	1.96 (1.60–2.40)	1.96 (1.13–3.38)	0.46 (0.39–0.54)
Others	0.97 (0.81–1.15)	0.81 (0.69–0.96)	1.48 (1.19–1.84)	0.62 (0.35–1.14)	0.73 (0.61–0.88)
p-value	<0.001	<0.001	<0.001	0.002	0.036
*Marital status*					
Never married	1	1	1	1	1
Currently married	2.11 (1.86–2.41)	5.15 (4.53–5.87)	7.09 (6.04–8.32)	0.58 (0.38–0.87)	0.34 (0.30–0.39)
Divorced/widowed	2.11 (1.71–2.62)	5.20 (4.22–6.41)	1.68 (1.33–2.12)	1.03 (0.59–1.81)	0.55 (0.44–0.68)
p-value	<0.001	<0.001	<0.001	0.771	<0.001

Values are odds ratios (95% confidence intervals)

Compared to men aged 15–29 years, those 45–59 years old were less likely to have had their sexual debut at less than 18 years (OR 0.20, 95% CI 0.17 to 0.25) and more likely to have used a condom in the last sex with a non-spousal non-cohabiting partner (OR 9.28, 95% CI 7.04 to 12.24). However, the oldest men (45–59 years) were more likely to have had at least two concurrent sex partners in the last 12 months (OR 1.32, 95% CI 1.11 to 1.56) and more than five lifetime partners (OR 4.43, 95% CI 3.88 to 5.06). Concerning transactional sex, the 45–59 year old men were less likely to have ever paid for sex (OR 0.17, 95% CI 0.06 to 0.47) but this was not a consistent trend with advancing age (P for trend = 0.154). Compared to men from the rural area, men from urban areas started sexual activity earlier (OR 1.37, 95% CI 1.22 to 1.54), were less likely to use a condom in sex with a non-spousal non-cohabiting partner (OR 0.31, 95% CI 0.26 to 0.36), and more likely to have had at least two concurrent sex partners in the last 12 months (OR 1.18, 95% CI 1.05 to 1.33) and more than five lifetime partners (OR 1.27, 95% CI 1.14 to 1.42). There was no difference in purchase of sexual services. Compared to Christians, Muslims (OR 0.46, 95% CI 0.39 to 0.54) were less likely to have had coital debut at an early age (P for trend = 0.036), more likely to use a condom in sex with a non-spousal non-cohabiting partner (OR 1.96, 95% CI 1.60 to 2.40), and less likely to have either concurrent (OR 0.69, 95% CI 0.59 to 0.82) or multiple lifetime (OR 0.47, 95% CI 0.40 to 0.54) sex partners. However, Muslims were more likely than Christians to pay for sex (OR 1.96, 95% CI 1.13 to 3.38). Men practising other religions were less likely to start sexual activity early (OR 0.73, 95% CI 0.61 to 0.88), more likely to use a condom in sex with a non-spousal non-cohabiting partner (OR 1.48, 95% CI 1.19 to 1.84) and less likely to have more than five lifetime partners (OR 0.81, 95% CI 0.69 to 0.96) compared to Christians. There was no difference between the two religious groups with regards to having concurrent sex partners in the previous 12 months and paying for sex. Compared to men who have never married, the married men were less likely to have paid for sex (OR 0.58, 95% CI 0.38 to 0.87) and more likely to have used a condom in the last sex with a non-spousal non-cohabiting partner (OR 7.07, 95% CI 6.04 to 8.32), had at least two concurrent sex partners in the last 12 months (OR 2.11, 95% CI 1.86 to 2.41), and more than five lifetime partners (OR 5.15, 95% CI 4.53 to 5.84). The pattern of risk behaviour was similar for divorced or widowed men compared to their counterparts who have never married.

### Multivariate analyses

Table 3 shows the adjusted odds ratios from the multiple logistic regression modelling. After controlling for marital status, place of residence, religion, and age, wealth and education remained significantly associated with unsafe sexual behaviours. Compared to poor men, the wealthiest men were less likely to use a condom in the last sex with a non-spousal non-cohabiting partner (OR 0.43, 95% CI 0.32 to 0.56) and more likely to have had at least two concurrent (OR 1.38, 95% CI 1.12 to 1.19) and more than five lifetime (OR 1.97, 95% CI 1.60 to 2.43) sex partners. There was no significant association between being wealthy and either age of coital debut or paying for sex. Compared to men who had never been to school, those with secondary or higher education were more likely to have started sex early (OR 2.73, 95% CI 2.10 to 3.56), less likely to have used a condom in the last sex with a non-spousal non-cohabiting partner (OR 0.24, 95% CI 0.16 to 0.38), and more likely to have had more than five lifetime partners (OR 2.59, 95% CI 2.02 to 3.31). In addition, there was a significant trend towards multiple concurrent sexual partnerships with increasing level of education (P for trend = 0.002).

**Table 3:** Adjusted odds ratios of the association between selected characteristics and sexual behaviours

**Characteristic**	**Had at least two sex partners in the last 12 months (Yes/No)**	**Had more than five lifetime sex partners (Yes/No)**	**Used condom in last sex with non-spousal non-cohabiting partner (Yes/No)**	**Has ever paid for sex (Yes/No)**	**Coital debut at 17 years or younger (Yes/No)**
*Wealth index*					
Low	1	1	1	1	1
Middle	1.16 (0.98–1.37)	1.73 (1.47–2.07)	0.57 (0.45–0.72)	0.82 (0.46–1.46)	1.05 (0.88–1.26)
High	1.38 (1.12–1.69)	1.97 (1.60–2.43)	0.43 (0.32–0.56)	0.47 (0.24–0.93)	1.04 (0.84–1.29)
p-value	0.043	<0.001	0.009	0.270	0.757
*Education*					
No school	1	1	1	1	1
Primary	0.93 (0.73–1.17)	2.20 (1.76–2.77)	0.49 (0.32–0.77)	1.68 (0.66–4.34)	2.07 (1.61–2.65)
Secondary/higher	1.19 (0.94–1.52)	2.59 (2.02–3.31)	0.24 (0.16–0.38)	1.10 (0.41–3.00)	2.73 (2.10–3.56)
p-value	0.002	<0.001	0.001	0.111	<0.001
*Age (years)*					
15–29	1	1	1	1	1
30–44	1.12 (0.96–1.32)	2.89 (2.45–3.40)	1.96 (1.61–2.39)	1.03 (0.63–1.67)	0.51 (0.43–0.59)
45–59	0.87 (0.72–1.07)	4.06 (3.29–5.02)	3.90 (2.86–5.30)	0.20 (0.07–0.61)	0.30 (0.24–0.36)
p-value	0.250	0.001	<0.001	0.111	0.001
*Residence*					
Rural	1	1	1	1	1
Urban	0.98 (0.84–1.15)	0.97 (0.82–1.14)	0.65 (0.53–0.80)	2.10 (1.24–3.56)	0.97 (0.82–1.14)
p-value	0.584	0.124	<0.001	0.024	0.665
*Religion*					
Christians	1	1	1	1	1
Muslims	0.74 (0.61–0.89)	0.51 (0.42–0.61)	0.95 (0.73–1.23)	2.12 (1.11–4.03)	0.68 (0.56–0.82)
Others	1.00 (0.83–1.20)	0.81 (0.67–0.98)	1.06 (0.82–1.36)	0.65 (0.35–1.23)	0.87 (0.71–1.05)
p-value	0.600	<0.001	0.484	0.485	0.005
*Marital status*					
Never married	1	1	1	1	1
Currently married	2.26 (1.91–2.67)	3.41 (2.89–4.02)	3.49 (2.86–4.25)	0.62 (0.38–1.03)	0.68 (0.58–0.81)
Divorced/widowed	2.11 (1.69–2.65)	3.61 (2.87–4.53)	1.18 (0.91–1.54)	1.08 (0.59–2.00)	0.88 (0.70–1.11)
p-value	<0.001	<0.001	0.300	0.876	0.665

Values are odds ratios (95% confidence intervals)

Other independent predictors of sexual behaviour were age, place of residence, religion, and marital status. Compared to young men (15–29 years), men in the oldest age group (45–59 years) were less likely to have had coital debut at younger than 18 years (OR 0.30, 95% CI 0.24 to 0.36), more likely to have used a condom in the last sex with a non-spousal non-cohabiting partner (OR 3.90, 95% CI 2.86 to 5.30) and less likely to have paid for sex (OR 0.20, 95% CI 0.07 to 0.61), but more likely to have had more than five lifetime sex partners (OR 4.06, 95% CI 3.29 to 5.02). Concerning place of residence, men living in urban areas were less likely to have used a condom in the last sex with a non-spousal non-cohabiting partner (OR 0.65, 95% CI 0.53 to 0.80) and more likely to have paid for sex (OR 2.10, 95% CI 1.24 to 3.56). For religion, Muslims were less likely to have started sex early (OR 0.68, 95% CI 0.56 to 0.82), had multiple concurrent sex partners in the last 12 months (OR 0.74, 95% CI 0.61 to 0.89), or multiple lifetime sex partners (OR 0.51, 95% CI 0.42 to 0.61), but more likely to have paid for sex (OR 2.12, 95% CI 1.22 to 4.03). Compared to never married men, married men were less likely to have had first sex at less than 18 years (OR 0.68, 95% CI 0.58 to 0.81) and more likely to have used condoms in the last sex with a non-spousal non-cohabiting partner (OR 3.49, 95% CI 2.86 to 4.25), but more likely to have had at least two concurrent sex partners in the last 12 months (OR 2.26, 95% CI 1.91 to 2.67).

## Discussion

This study examined the association between wealth and sexual behaviour among resident Cameroonian men. Compared to the poorer men, wealthier men were more likely to start sexual activity at an early age, and have unprotected sex with a non-spousal non-cohabiting partner and both multiple concurrent and multiple lifetime sex partners. Other investigators have obtained similar results in other settings [18,19,27]. Mitsunaga and colleagues reported in 2005 that wealthy men in Nigeria are more likely to engage in extramarital sex than their poorer counterparts [27]. However, the findings are not consistent with those of others [13,28]. Kimuna and Djamba reported in 2005 that none of three proxies of wealth (education, occupation and household wealth index) was associated with extramarital sex in Zambia [28]. These differences may be due to differences in socio-cultural practices and the different stages of the HIV epidemic in different countries.

We also found that unsafe sexual behaviours became significantly more common with increasing level of education. Kirunga and Ntozi reported similar findings in 1997 in the Rakai district of Uganda [29]. However, a previous study in the city of Yaoundé reported that educated men were more prone to adopt safe sexual behaviours [30]. De Walgue and colleagues reported that although the risk for HIV infection increased with the level of education in south-western Uganda in 1989/1990, the trend reversed over the following decade such that in 1999/2000 the risk of HIV decreased with increasing education [31]. Paasche-Orlow and colleagues also reported in 2005 that educational attainment was associated with lower HIV risk sexual behaviours [32]. These differences suggest that the situation is not static, but changes over time. In the same country, different regions may be at different stages in the transition from unsafe to safer sexual behaviours.

Unsafe sexual behaviours were more prevalent in urban than rural areas. This is consistent with previous reports [33,34]. However, Voeten and colleagues have reported a higher prevalence of unsafe sexual behaviours in the rural compared to the urban areas of Nyanza province in Kenya [35]. Hladjk and colleagues have also reported the expansion of the HIV epidemic to rural areas and a trend towards a decline in some cities [36]. This suggests that like for education, different regions of the same country might be at different stages of the implementation of HIV prevention strategies. We found a higher prevalence of unsafe sexual behaviours in married and formerly married men; consistent with the findings of some [37,38] but not all authors [39]. These differences might be explained by cultural differences and the differences in the stages of the HIV epidemic. We found that there were more unsafe sexual behaviours among Christians than Muslims. The 2004 Cameroon DHS showed that animists have a lower HIV prevalence (1.2%) than Muslims (4.5%), Catholics (5.9%), and Protestants (6.3%) [25]. Other investigators have found that religiosity was positively associated with unsafe sexual behaviours among injecting drug users [40]. However, McCree and colleagues reported that religious African-American adolescent girls are less likely to engage in unsafe sexual behaviours [41]. Religion is strongly embedded in cultures and the degree of religiosity tends to vary from country to country.

Our study has some limitations. We did not control for unsafe health care practices. Previous epidemiological analyses of currently available data have suggested that the HIV epidemic in sub-Saharan Africa may be predominantly driven by iatrogenic transmission through unsafe injections [2,4,20,21]. The authors argue that when unsafe health care is controlled for, the apparent associations between sexual behaviours and HIV infection become non-significant [3,22]. Data from the 2004 Cameroon DHS do point to some iatrogenic transmission of HIV [25]. HIV prevalence among single men who have never had sex was 1.0%, and the prevalence of HIV was higher among men who reported having used condoms in the previous 12 months than in those who did not (5.7% versus 4.7%). However, HIV infection among condom users is not necessarily acquired through a non-sexual route; because for condoms to be effective in reducing (though not eliminating) the risk of HIV infection, they must be used correctly and consistently [42]. Certainly some infections in Cameroon and elsewhere are transmitted by unsafe injections, but we are of the opinion that current epidemiological evidence is overwhelmingly in favour of a predominant sexual HIV epidemic in sub-Saharan Africa [1]. Another limitation is that information was not collected on the sex of partners nor whether the men have ever practised anal sex. While sexual transmission in the context of sub-Saharan Africa may indirectly infer transmission during penile-vaginal intercourse, anal intercourse (whether homosexual or heterosexual) is not rare in the region [43,44]. We did not examine the potential confounding effect of polygamy on the relationships found in this study because less than 10% of participants had two or more wives. Nevertheless, the 2004 Cameroon DHS data did not find an association between type of matrimonial union and HIV prevalence (5.5% in polygamous and 5.4% in monogamous men) [25].

## Conclusion

In conclusion, we found that wealthy men in Cameroon (as measured by household amenities and educational attainment) are more likely to start sexual activity early and have both multiple concurrent and lifetime sex partners, and less likely to (consistently) use a condom in sex with a non-spousal non-cohabiting partner. These unsafe sexual behaviours may explain the higher HIV prevalence among wealthier men in the country. While these findings do not suggest a redirection of HIV prevention efforts from the poor to the rich, they do call for efforts to ensure that HIV prevention messages get across all strata of society.

## Competing interests

The author(s) declare that they have no competing interests.

## Authors' contributions

EJK and CSW conceived the study, collected the data, did the analyses and interpretation, and wrote the first draft of the manuscript. REM, PN, and LK critically revised the manuscript for important intellectual content. All authors read and approved the final manuscript.

## Pre-publication history

The pre-publication history for this paper can be accessed here:



## References

[B1] UNAIDS Report of the global AIDS epidemic: May 2006. http://www.unaids.org/en/HIV_data/2006GlobalReport/default.asp.

[B2] Brody S (2004). Declining HIV rates in Uganda: due to cleaner needles, not abstinence or condoms. Int J of STD AIDS.

[B3] Gisselquist D, Potterat JJ, Brody S, Vachon F (2003). Let it be sexual: how health care transmission of AIDS in Africa was ignored. Int J STD AIDS.

[B4] Brewer DD, Brody S, Drucker E, Gisselquist D, Minkin SF, Potterat JJ, Rothenberg RB, Vachon F (2003). Mounting anormalies in the epidemiology of HIV in Africa: cry the beloved paradigm. Int J STD AIDS.

[B5] Kallestrup P, Zinyama R, Gomo E, Gerstoft J, Ullum H (2004). HIV in Africa – still a matter of unsafe sex. Int J STD AIDS.

[B6] Boily MC, White RG, Alary M, Lowndes CM, Orroth K (2004). Transmission of HIV via unsafe injection or unsafe sex? Anomalies or misunderstanding?. Int J STD AIDS.

[B7] Schmid GP, Buve A, Mugyenyi P, Garnett GP, Hayes RJ, Williams BG, Calleja JG, De Cock KM, Whitworth JA, Kapiga SH (2004). Transmission of HIV-1 infection in sub-Saharan Africa and effect of elimination of unsafe injections. Lancet.

[B8] Halperin DT, Steiner MJ, Cassell MM, Green EC, Hearst N, Kirby D, Gayle HD, Cates W (2004). The time has come for common ground on preventing sexual transmission of HIV. Lancet.

[B9] Nyindo M (2005). Complementary factors contributing to the rapid spread of HIV-I in sub-Saharan Africa: a review. East Afr Med J.

[B10] Volk JE, Prestage G, Jin F, Kaldor J, Ellard J, Kippax S, Grulich AE (2006). Risk factors for HIV seroconversion in homosexual men in Australia. Sex Health.

[B11] Mekonnen Y, Sanders E, Messele T, Wolday D, Dorigo-Zestma W, Schaap A, Mekonnen W, Meless H, Mihret W, Fontanet A, Coutinho RA, Dukers NH (2005). Prevalence and incidence of, and risk factors for, HIV-1 infection among factory workers in Ethiopia, 1997–2001. J Health Popul Nutr.

[B12] Todd CS, Khakimov MM, Alibayeva G, Abdullaeva M, Giyasova GM, Saad MD, Botros BA, Bautista CT, Sanchez JL, Carr JK, Earhart KC (2006). Prevalence and Correlates of Human Immunodeficiency Virus Infection Among Female Sex Workers in Tashkent, Uzbekistan. Sex Transm Dis.

[B13] Mboto CI, Davies A, Fielder M, Jewell AP (2006). Human immunodeficiency virus and hepatitis C co-infection in sub-Saharan West Africa. Br J Biomed Sci.

[B14] Andrews G, Skinner D, Zuma K (2006). Epidemiology of health and vulnerability among children orphaned and made vulnerable by HIV/AIDS in sub-Saharan Africa. AIDS Care.

[B15] Xia Q, Osmond DH, Tholandi M, Pollack LM, Zhou W, Ruiz JD, Catania JA (2006). HIV prevalence and sexual risk behaviors among men who have sex with men: results from a statewide population-based survey in California. J Acquir Immune Defic Syndr.

[B16] Kalichman SC, Simbayi LC, Kagee A, Toefy Y, Jooste S, Cain D, Cherry C (2006). Associations of poverty, substance use, and HIV transmission risk behaviors in three South African communities. Soc Sci Med.

[B17] Kalichman SC, Simbayi LC, Jooste S, Cherry C, Cain D (2005). Poverty-related stressors and HIV/AIDS transmission risks in two South African communities. J Urban Health.

[B18] Shelton JD, Cassell MM, Adetunji J (2005). Is poverty or wealth at the root of HIV?. Lancet.

[B19] Fenton L (2004). Preventing HIV/AIDS through poverty reduction: the only sustainable solution?. Lancet.

[B20] Becker ML, Ramesh B, Moses S, Blanchard JF (2006). Response to Brewer et al regarding our article 'Association between medical injections and HIV infection in a community based study in India'. AIDS.

[B21] Brewer DD, Potterat JJ, Brody S (2006). Research design determines what can be known about modes of HIV transmission. AIDS.

[B22] Deutchert E, Brody S (2006). The role of health care in the spread of HIV/AIDS in Africa: Evidence from Kenya. Int J STD AIDS.

[B23] Hawkes K (1994). On life-history evolution. Current Anthropology.

[B24] Cashdan E (1996). Women's mating strategies. Evolutionary Anthropology.

[B25] National Institute of Statistics (NIS) and ORC Macro (2004). *Cameroon Demographic and Health Survey 2004*. Calverton Maryland USA: NIS and ORC Macro.

[B26] Gwatkin DR, Rustein S, Johnson K, Pande R, Wagstaff A, For the HNP/Poverty Thematic Group of the World Bank (2000). *Socio-economic differences in health, nutrition and population in Cameroon*.

[B27] Mitsunaga TM, Powell AM, Heard NJ, Larsen UM (2005). Extramarital sex among Nigerian men: polygyny and other risk factors. J Acquir Immune Defic Syndr.

[B28] Kimuna S, Djamba Y (2005). Wealth and extramarital sex among men in Zambia. Int Fam Plan Perspect.

[B29] Kirunga CT, Ntozi JP (1997). Socio-economic determinants of HIV serostatus: a study of Rakai District, Uganda. Health Transit Rev.

[B30] Glynn JR, Carael M, Buve A, Anagonou S, Zekeng L, Kahindo M, Musonda R (2004). Study Group on Heterogeneity of HIV Epidemics in African Cities. Does increased general schooling protect against HIV infection? A study in four African cities. Trop Med Int Health.

[B31] de Walque D, Nakiyingi-Miiro JS, Busingye J, Whitworth JA (2005). Changing association between schooling levels and HIV-1 infection over 11 years in a rural population cohort in south-west Uganda. Trop Med Int Health.

[B32] Paasche-Orlow MK, Clarke JG, Hebert MR, Ray MK, Stein MD (2005). Educational attainment but not literacy is associated with HIV risk behavior among incarcerated women. J Womens Health (Larchmt).

[B33] Springer AE, Selwyn BJ, Kelder SH (2006). A descriptive study of youth risk behavior in urban and rural secondary school students in El Salvador. BMC Int Health Hum Rights.

[B34] Mnyika KS, Klepp KI, Kvale G, Ole-Kingori N (1997). Determinants of high-risk sexual behaviour and condom use among adults in the Arusha region, Tanzania. Int J STD AIDS.

[B35] Voeten HA, Egesah OB, Habbema JD (2004). Sexual behavior is more risky in rural than in urban areas among young women in Nyanza province, Kenya. Sex Transm Dis.

[B36] Hladik W, Shabbir I, Jelaludin A, Woldu A, Tsehaynesh M, Tadesse W (2006). HIV/AIDS in Ethiopia: where is the epidemic heading?. Sex Transm Infect.

[B37] Bakhireva LN, Abebe Y, Brodine SK, Kraft HS, Shaffer RA, Boyer CB (2004). Human immunodeficiency virus/acquired immunodeficiency syndrome knowledge and risk factors in Ethiopian military personnel. Mil Med.

[B38] Bassett MT, McFarland WC, Ray S, Mbizvo MT, Machekano R, van de Wijgert JH, Katzenstein DA (1996). Risk factors for HIV infection at enrollment in an urban male factory cohort in Harare, Zimbabwe. J Acquir Immune Defic Syndr Hum Retrovirol.

[B39] Quigley MA, Morgan D, Malamba SS, Mayanja B, Okongo MJ, Carpenter LM, Whitworth JA (2000). Case-control study of risk factors for incident HIV infection in rural Uganda. J Acquir Immune Defic Syndr.

[B40] Hasnain M, Sinacore JM, Mensah EK, Levy JA (2005). Influence of religiosity on HIV risk behaviors in active injection drug users. AIDS Care.

[B41] McCree DH, Wingood GM, DiClemente R, Davies S, Harrington KF (2003). Religiosity and risky sexual behavior in African-American adolescent females. J Adolesc Health.

[B42] Bracher M, Santow G, Watkins SC (2004). Assessing the potential of condom use to prevent the spread of HIV: a microsimulation study. Stud Fam Plann.

[B43] Brody S, Potterat JJ (2003). Assessing the role of anal intercourse in the epidemiology of AIDS in Africa. Int J STD AIDS.

[B44] Lane T, Pettifor A, Pascoe S, Fiamma A, Rees H (2006). Heterosexual anal intercourse increases risk of HIV infection among young South African men. AIDS.

